# Salicylic Acid Manipulates Ion Accumulation and Distribution in Favor of Salinity Tolerance in *Chenopodium* *quinoa*

**DOI:** 10.3390/ijerph19031576

**Published:** 2022-01-29

**Authors:** Hamid Mohammadi, Bahareh Rahimpour, Hadi Pirasteh-Anosheh, Marco Race

**Affiliations:** 1 Faculty of Agriculture, Azarbaijan Shahid Madani University, Tabriz 5375171379, Iran; hmohammadi@azaruniv.ac.ir (H.M.); baharehrahimpour45@gmail.com (B.R.); 2 National Salinity Research Center, Agricultural Research, Education and Extension Organization, Yazd 8917357676, Iran; 3 Department of Civil and Mechanical Engineering, University of Cassino and Southern Lazio, 03043 Cassino, Italy; marco.race@unicas.it

**Keywords:** haloculture, halophyte, mechanism, salinity tolerance, selectivity, storage factor

## Abstract

Although the effects of salicylic acid (SA) on increasing plant growth in saline conditions have been well known, the mechanisms of induction of salinity tolerance, especially in quinoa (*Chenopodium quinoa* Willd.), are not fully understood. In the present work, two quinoa genotypes (Titicaca and Giza1) were treated with different SA concentrations (0, 0.75, and 1.5 mM) under varied irrigation water salinities (0, 7, 14, and 21 dS m^−1^). Salinity decreased shoot and root growth, potassium (K^+^) concentration, and potassium to sodium ratio (K/Na) and increased sodium (Na^+^) and chlorine (Cl^−^) concentrations in both cultivars. Calcium (Ca^2+^) and magnesium (Mg^2+^) concentrations increased in 7 dS m^−1^ but decreased in higher salinities. The growth and salinity tolerance of Giza1 were higher, while the growth of Giza1 increased and of Titicaca decreased in high salinity. Salicylic acid at 0.75-mM concentration increased shoot and root growth and improved the ions concentration in favor of the plant, while the 1.5-mM concentration either had no significant effect or had a negative impact. The ions distribution estimated by K/Na selectivity and storage factor (SF) indicated quinoa accumulated more ions in roots under saline conditions. Salicylic acid increased NaSF, ClSF, and MgSF and decreased KSF and CaSF, meaning less Na^+^, Cl^−^, and Mg^2+^ and more K^+^ and Ca^2+^ transferred to shoots in SA-treated plants. Importantly, Giza1, as the more tolerant cultivar, had higher NaSF and ClSF and lower KSF, CaSF, and MgSF. In general, the concentrations of ions in roots were higher than in shoots. The results indicated more ions accumulation in the root could be one of the most important mechanisms of salinity tolerance in quinoa, and the more tolerant cultivar (Giza1) transferred less Na^+^ and Cl^−^ and more K^+^ and Ca^2+^ and Mg^2+^ to the shoot.

## 1. Introduction

The excessive pumping of groundwater for irrigation of agricultural fields together with the lack or decrease of natural recharge is leading to seawater intrusion in arid and semi-arid regions [1,2]. Groundwater salinization may have unexpected cascading consequences and crises on social, economic, and environmental systems [3]. For example, the increasing salinity of groundwater may lead to an increase in soil salinity together with a sodium (Na^+^) accumulation. As a consequence, a decrease in the yield of agricultural production could be observed, with dramatic consequences for the population. In addition, the Na^+^ accumulation in the soil, with a consequent increase of the SAR, can decrease the permeability of agricultural soils and, consequently, may further slowdown the natural groundwater recharge [4]. A practical approach to increase plant production for providing food security for the growing world population is the use of salinity tolerant plants, such as halophytes [5,6]. In particular, the adoption of halophytes may also reduce the Na^+^ accumulation in the soil, thus avoiding the above-mentioned problems [7]. A halophyte is a plant with the ability to adapt in saline conditions through preventing salt from entering the plant or reducing the salt concentration in the cytoplasm [2].

Salinity usually prevents plant growth in two different ways: osmotic stress and ion toxicity [8]. The first is enhanced osmotic stress as an early reaction, which makes it difficult for the plant to absorb water, and the second is ion toxicity as the late reaction. The ion toxicity is due to the effect of salt, such as Na^+^ and Cl^−^ ions, on changes in cellular function, which reduces nutrient uptake, enzyme activity, photosynthesis, and metabolism [9]. The initial phase of salt stress is due to the effect of salt outside the root zone, which prevents water uptake, root growth, cell shrinkage, and leaf growth and reduces new leaves and damages cells in wet leaves, etc., while salt stress in the late phase is the result of the toxic effect of salt inside the plant [10].

Most common plants do not have high salinity tolerance [5]. The presence of salt in the soil has adverse effects on the growth and development of these plants at morphological, physiological, and biochemical levels [11]. Releasing plants that tolerate salinity stress well, although with slight changes in the genetic map, is very difficult because tolerance to salinity stress is a multi-gene trait [12]. On the other hand, the use of saline water as irrigation water is an inevitable necessity due to limited freshwater resources. Therefore, halophytes should be used as high-yielding crops in saline conditions [9].

Halophytes are introduced as plants that adapt to and grow in soils with high salt concentrations [12]. Therefore, halophytes can be considered as an ideal model for understanding the complex physiological and genetic mechanisms of salt-stress tolerance. Although about two percent of known plant species are halophytes, they are rarely used for food and forage [6,13]. Quinoa (*Chenopodium quinoa* C.L. Willdenow (Willd.)) is one of the high potential halophytes as a human food source and a forage for livestock feeding, which, with a high nutritional quality, has a high tolerance to salinity [14].

Quinoa is a plant of the Amaranthaceae family, C_3_, and facultative halophyte. In general, plants adapt to growing in high-salinity conditions via three salt tolerance mechanisms: reduction in plant Na^+^ intake, Na^+^ accumulation in vacuole and excretion of adsorbed Na^+^ [12], or three salinity avoidance mechanisms, namely secretions, leaf shedding, and increased plant water content [15]. Quinoa has various mechanisms to deal with salt stress. One of these mechanisms is a change in ion distribution and salt exclusion through the salt bags on the surface and under the leaves and on the panicle, which causes the excretion of excess absorbed salt [14]. Quinoa genotypes vary widely in terms of maturity, photoperiod sensitivity, and salinity tolerance. Therefore, selecting the appropriate genotype plays an important role in crop success [16].

One of the reasons for the decrease in growth and yield of quinoa under salinity stress is a change in the balance of phytohormones [6,17]. The use of plant-growth regulators (PGR) significantly causes hormonal balance in plants in saline conditions and thus increases the plant tolerance to salt stress [11]. Salicylic acid (SA) is one of the most important PGRs that regulates germination, yield, glycolysis, and flowering and promotes plant tolerance and improves growth and yield [1]. Studies show that SA regulates unilateral internal K^+^ channel inactivation, which in turn plays a role in ionic balance, aperture closure, photosynthesis, enzymatic activity, improvement of protein and carbohydrate synthesis, and product quality [8]. Because SA also plays a role in regulating redox, high concentrations of SA may reduce stress tolerance through impaired redox status [8,17]. Hence, determining the optimal concentration in this regard is very important because reduced growth and yield of different plants have been reported at high concentrations of SA [17].

Although important research has been done on the effect of SA on the growth and physiology of various plants under saline conditions, its role in ion distribution and related tolerance mechanisms is not fully understood. Furthermore, there is little knowledge on the mechanisms of SA in induction of salinity tolerance, especially in quinoa. Therefore, considering the promising potential of quinoa in providing human food and high-salt and drought-stresses tolerance, this study was conducted to investigate the role of SA in improving salinity tolerance through ion distribution (Na^+^, K^+^, Cl^−^, Ca^2+^, Mg^2+^) in shoots and roots of two quinoa genotypes.

## 2. Materials and Methods

### 2.1. Experimental Procedure

This research was done in a factorial experiment based on randomized complete block design (RCBD) with three replications at Azarbaijan Shahid Madani University in 2019–2020. The treatments were four salinity levels: 0 (as control), 7, 14, and 21 dS m^−1^; two exogenous SA applications: 0.75 and 1.5 mM, along with a control (no-SA); and two quinoa cultivars: Titicaca and Giza1.

Ten uniform and intact seeds were sown in each 18-L pot, whose emerged seedlings were thinned to 5 plants per pot. The pots were kept in a controlled environment with the minimum and the maximum temperatures about 14 °C and 28 °C, respectively; relative humidity was about 55–60% and the day-length 14 h (using both fluorescent and incandescent lamps).

### 2.2. Treatments

Salinity treatments were applied by irrigation of pots with varied electrical conductivity (EC), which were made using sodium chloride (NaCl). The EC of irrigation water (ECiw) was controlled by a portable EC-meter. To prevent sudden stress, the plants were gradually exposed to saline treatment. In each irrigation, the EC of drainage water of the pots was monitored in order to keep EC of the potting soil constant and ensure the correctness of applied salinity treatments.

After applying salinity treatments, the SA solutions were made using distilled water and ethanol. To dissolve the SA in water more easily, the beaker containing solutions was placed on a magnetic heater. Salicylic acid solutions were sprayed on shoots of the plant in the early hours of a sunny day without wind. To prevent sediment, the solution was stirred continuously during foliar application. Titicaca and Giza1 cultivars originate from Denmark and Egypt, respectively. The thousand-grain weight, plant height, protein content, and saponin in Titicaca are 2.5–3.0 g, 80–100 cm, 12–15%, and 2–2.5%, respectively, and in Giza1 are 2.5–3.0 g, 100–120 cm, 10–13%, and 2.2–2.5%, respectively.

### 2.3. Measurements

The 70-day-old quinoa plants were completely harvested, and the shoot and root sections were separated. The samples were kept in a ventilated oven at 70 ± 2 °C for 48 h, and then, the shoot dry weight (SDW) and root dry weight (RSW) were measured with a digital scale. To measure the concentration of Na^+^, K^+^, Cl^−^, Ca^2+^, and Mg^2+^ ions in quinoa plants, the dried samples were completely ground and turned to ashes in a furnace at 600 °C. The concentrations of Na^+^ and K^+^ were measured by using a flame photometer, and the concentration of Cl^−^ was obtained by titration using the chloride ion electrode. Furthermore, the concentrations of Ca^2+^ and Mg^2+^ in the samples were determined using an atomic absorption spectrometer. The ratio of concentration of K^+^ to Na^+^ was considered as K/Na.

The concentrations were considered as the ion accumulations, and to quantify the ion distribution, two indicators of K^+^ to Na^+^ selectivity and storage factor (SF) were used. Using the K/Na, the plant ion selectivity index (SI) was calculated [18] as following:

$$\mathrm{SI}=\frac{ShootK^{+}/Na^{+}}{RootK^{+}/Na}\;$$

As a new indicator, SF was defined to determine the distribution of ions between shoots and roots. The SF for the all ions was also obtained based on the following equation [19]:

$$\mathrm{SF}=\frac{RC_{i}}{\left(RC_{i}+SC_{i}\right)}$$

where *RC_i_* and *SC_i_* are the concentration of each ion (i) in root and shoot, respectively. Indeed, SF quantified the ratio of accumulated ions in root to the total adsorbed ions by plants.

### 2.4. Data Analysis

Correlation and regression analyses were used to determine the relationship between the traits. The relationships between ions concentration and SDW, RDW, and total dry weight (SDW^+^RDW) were estimated by the Pearson correlation. Additionally, the most effective traits on SDW and RDW were identified through stepwise regression. The statistical analyses including analysis of variance (ANOVA), means comparison using the least significant difference (LSD) test, correlation, and stepwise regression were performed with SAS software version 9.4.

## 3. Results

### 3.1. Shoot and Root Dry Weight

**Figure 1:**
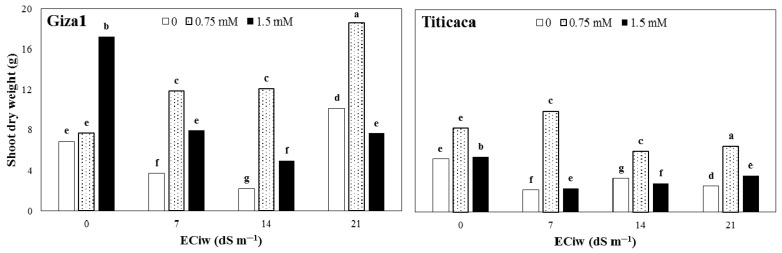
Shoot dry weight of two quinoa cultivars treated by varied salicylic acid concentrations grown under different salinity of irrigation water (ECiw). The columns with at least a similar letter in each figure are not significantly different based on LSD at 1% probability level.

The results showed that in non-saline and no-SA conditions, shoot dry weight in Giza1 cultivar (6.89 g) was higher than Titicaca (5.26 g) (Figure 1). In Titicaca, salinity had a negative effect on shoot dry weight so that shoot weight decreased with increasing salinity concentration at all levels. However, the trend of changes was slightly different for Giza1. Shoot dry weight initially decreased with increasing salinity from 0 to 14 dS m^−1^, while it showed a significant increase with increasing salinity to 21 dS m^−1^, as this amount (10.14 g) was the highest shoot dry weight for both cultivars at different salinity levels (Figure 1). Furthermore, the effect of SA application in non-saline conditions was different on the two cultivars. With increasing SA concentration, shoot dry weight of Giza1 also increased and reached the height value (17.2 g) at 1.5-mM concentration. However, in non-saline conditions, the highest amount of shoot dry weight (8.3 g) of Titicaca cultivar was obtained at 0.75 mM SA (Figure 1). The effect of SA application on shoot dry weight changes was the same in higher salinity levels, so the highest value was obtained at a concentration of 0.75 mM in both cultivars. In non-saline and high salinity levels, shoot dry weight was higher for Giza1 than Titicaca cultivar (Figure 1).

**Figure 2:**
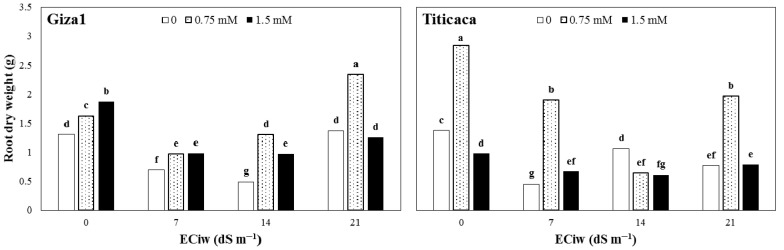
Root dry weight of two quinoa cultivars treated by varied salicylic acid concentrations grown under different salinity of irrigation water (ECiw). The columns with at least a similar letter in each figure are not significantly different based on LSD at 1% probability level.

The mean comparisons showed that in contrast to shoots, root dry weight of Titicaca was greater than Giza1 cultivar by 5.4% in non-saline conditions and no-SA application (Figure 2). Salinity reduced root dry weight of both cultivars up to 14 dS m^−1^ salinity; however, the root dry weight of both cultivars, especially Giza1, was increased in 21 dS m^−1^ (Figure 1). In non-saline conditions, the amount and mode of effectiveness of SA application on root dry weight was different for the two cultivars. In Giza1 cultivar, increasing the concentration of SA increased the root dry weight, and the highest amount was observed in the 1.5-mM concentration. However, in Titicaca cultivar, 1.5-mM SA concentration had a negative effect on root dry weight, and the highest root dry weight was obtained at 0.75-mM concentration (Figure 2). The effect of SA application on root dry weight under salinity stress was the same as non-saline conditions. In general, with increasing salinity stress, application of 1.5 mM SA had a negative effect on root dry weight of both cultivars, while 0.75 mM increased root dry weight (Figure 2).

### 3.2. Ion Concentration in Shoot

Comparisons of means showed that Na^+^ accumulation in shoots was increased with increasing salinity (Table 1). The highest amount of shoot Na^+^ concentration was obtained with a 21-dS m^−1^ salinity with 4.36-times increase compared to the non-saline treatment. However, there was no significant difference in shoot Na^+^ concentration of 14 and 21 dS m^−1^ salinities (Table 1). Foliar application of SA resulted in a significant reduction in Na^+^ concentration; however, no significant difference was observed between 0.75- and 1.5-mM concentrations. The highest and lowest concentrations of Na^+^ were obtained at 0- and 0.75-mM SA concentration, respectively, as Na^+^ concentration was less in 0.75 mM SA than no-SA by 27% (Table 1).

With increasing salinity level, the K^+^ concentration of shoots decreased and reached the lowest value at 21-dS m^−1^ salinity (Table 1). In contrast of Na^+^, foliar application of SA caused a significant increase in K^+^ concentration. However, no significant difference was observed between 0.75- and 1.5-mM SA concentrations. The highest and lowest K^+^ concentrations were obtained at 0.75- and 0-mM SA concentrations so that K^+^ concentration was greater in no-SA than 0.75 mM SA by 20% (Table 1).

As salinity increased the concentration of Na^+^ and decreased K^+^, the potassium-to-sodium ratio (K/Na) of shoots was decreased in saline treatments (Table 1). The highest (2.25) and lowest (0.02) values of K/Na were obtained at non-saline and 21 dS m^−1^ salinity, respectively. The SA application enhanced K/Na in non-saline and saline conditions. The highest and lowest values of K/Na were obtained at concentrations of 1.5 mM and no-SA application (10.7% increase), respectively. In no-SA application, increasing salinity stress reduced K/Na up to a minimum in 21 salinity dS m^−1^. However, foliar application of SA at concentrations of 0.75 and 1.5 mM significantly increased K/Na. At all SA levels, the highest and lowest K/Na were obtained at non-saline and 21 dS m^−1^ salinities, respectively (Table 1).

The results also showed with increasing salinity, Cl^−^ concentration increased up to a highest value (1.1%) at 21-dS m^−1^ salinity (Table 2). Furthermore, SA foliar application reduced the Cl^−^ concentrations, and the lowest amount was obtained at 1.5-mM SA concentration (by 22.14% reduction compared to the no-SA). However, there was no significant difference between 0.75- and 1.5-mM concentrations in most cases (Table 1).

The response of Ca^2+^ concentration in quinoa to different levels of salinity stress had a non-uniform trend. Therefore, initially, with increasing salinity level from 0 to 7 dS m^−1^, Ca^2+^concentration increased by about 6%, while, with increasing salinity level to 14 and 21 dS m^−1^, the Ca^2+^ concentration was reduced, and the lowest value was obtained with a decrease of 27% in the 21 dS m^−1^ treatment (Table 1). Foliar application of SA had a clear-cut effect on Ca^2+^ concentration, and with increasing SA concentration, Ca^2+^ was also increased. The highest Ca^2+^ concentration was obtained in the 1.5-mM SA treatment (20.3% increase compared to the no-SA); however, no significant difference was observed between the concentrations of 0.75 and 1.5 mM SA (Table 1).

The concentration of Mg^2+^ varied in different cultivars, as it was higher in Titicaca than Giza’ (Table 1). The response of Mg^2+^ to salinity was similar to Ca^2+^. In general, Mg^2+^ concentration initially increased with increasing salinity to 7 dS m^−1^ and began to decrease from 14-dS m^−1^ salinity level. The highest (1.31%) and lowest (0.92%) Mg^2+^ concentration were obtained at 7 and 21 dS m^−1^ salinities, respectively.

### 3.3. Ion Concentration in Root

Concentrations of ions in quinoa root in response to salinity and SA treatments often behaved similarly to shoots (Table 1 and Table 2). The differences were mostly in the intensity of the changes and not in the overall trend. Based on mean comparisons, with increasing salinity stress, root Na^+^ and K^+^ concentration increased (Table 2). The highest concentrations of Na^+^ (4.16%) and K^+^ (3.71%) were observed in Giza1 plants grown under 21-dS m^−1^ salinity and treated by 1.5 mM SA foliar application. The lowest Na^+^ concentration was obtained in non-saline conditions without SA in Giza1 cultivar as 0.17%, while the lowest K^+^ concentration was observed in non-saline conditions with 1.5-mM SA application Titicaca cultivar as 0.42%. The K/Na of root was decreased with salinity stress, as Giza1 cultivar under non-saline condition had the highest K/Na, and the Titicaca cultivar under 14 dSm^−1^ had the lowest one (Table 2).

Magnesium concentration was increased with increasing salinity stress up to 7 dS m-1. Therefore, the highest amount of Mg^2+^ was related to Giza1 plants in 7-dS m^−1^ salinity with 0.75-mM SA treatments, and the lowest amount was related to the 21-dS m^−1^ salinity without SA application in Titicaca cultivar (Table 2). Furthermore, the highest (76.8%) and lowest (10.8%) concentrations of Cl^−^ were obtained in Titicaca cultivar at non-saline conditions with 1.5-mM SA application. Foliar application of SA had a significant effect on Ca^2+^ concentrations, and the highest (1.2%) and the lowest (1.1%) Ca^2+^ were obtained in no-SA and 1.5-mM SA foliar application (Table 2).

### 3.4. Ion Distribution

**Figure 3:**
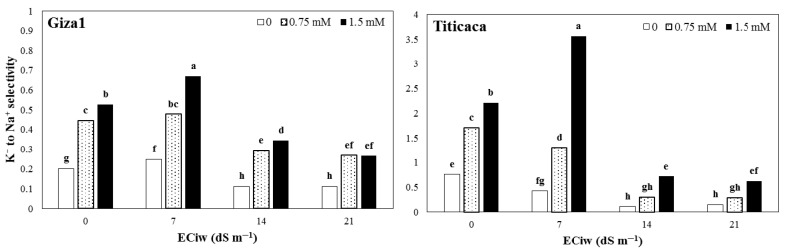
The K^+^ to Na^+^ selectivity of two quinoa cultivars treated by varied salicylic acid concentrations grown under different salinity of irrigation water (ECiw). The columns with at least a similar letter in each figure are not significantly different based on LSD at 1% probability level.

The results showed that the highest K^+^ to Na^+^ selectivity was obtained at salinity 7 dS m^−1^; however, higher salinity levels reduced the selectivity to less than the non-saline (Figure 3). On average, the selectivity indices of quinoa plants treated with 7.5 and 1.5 mM SA were higher than no-SA treatments by 2.4 and 2.4 times, respectively. In both non-saline conditions, application of SA increased K^+^ to Na^+^ selectivity of two cultivars. The highest amount of ion selectivity was observed at 7-dS m^−1^ salinity with the application of 7.5 mm SA in Titicaca, and the lowest value was related to 21-dS m^−1^ salinity and no-SA in Giza1 cultivar (Figure 3).

In general, SF was lower in salt-stressed plants so that the lowest NaSF, KSF, and MgSF were obtained in non-saline treatments, and ClSF and CaSF were also low in non-saline conditions (Table 3). The highest values of NaSF, KSF, ClSF, CaSF, and MgSF were obtained at salinity levels of 7, 21, 21, 7, and 21 m^−1^, respectively. Interestingly, the effects of SA on the SF of different ions were not similar. SA-treated plants had higher NaSF, ClSF, and MgSF and lower KSF and CaSF. The SF responses of different ions to salinity, and especially SA, were almost similar in the two quinoa cultivars, but their values were varied. Titicaca cultivar had higher NaSF and ClSF, while KSF, CaSF, and MgSF were higher in Giza1 cultivar (Table 3).

Based on correlation analysis (Table 4), shoot dry weight were significantly correlated with root K^+^ (+0.396 *), KSF (−0.531 **), shoot K/Na (+0.433 *), root K/Na (+0.413 *), ClSF (+0.403 *), shoot Mg^2+^ (−0.458 **), root Mg^2+^ (+0.473 *), and MgSF (+0.526 **). On the other hand, shoot Na^+^ (−0.407 *) and shoot K/Na (+0.426 *) had significant correlations with root dry weight. The correlation of shoot Na^+^ (−0.405 *), NaSF (+0.381 *), root K^+^ (+0.410 *), shoot K/Na (+0.624 **), root K/Na (+0.440 *), ClSF (+0.452 *), shoot Mg^2+^ (−0.402 **), root Mg^2+^ (+0.409 *), and MgSF (+0.444 *) was significant with total dry weight (shoots + roots) (Table 4).

The results of stepwise regression showed that MgSF, shoot Mg^2+^, Shoot K/Na, root K/Na, and ClSF were the most effective traits on shoot dry weight (Table 5). Furthermore, the most effective traits on root dry weight were root K/Na, shoot Na^+^, and NaSF (Table 5).

## 4. Discussion

The results achieved in this work showed that with increasing salinity level, shoot dry weight decreased in both quinoa cultivars. The reason for this decrease can be attributed to the limited supply of metabolites for growing tissues due to reduced water uptake capacity of roots and/or excessive increase of Na^+^ and Cl^−^ ions [13,18]. These ions cause significant physiological disorders. Under saline conditions, slower growth in the early stages of the stress period may be a compatible response by plants for survival, which allows them to store assimilates, repair damaged structures, and resume physiological functions [11]. Cai and Gao [20] showed that quinoa cultivars with lower biomass are more salinity tolerant, especially at lower salinity levels (less than 300 mM NaCl).

Although salt stress negatively affected both Titicaca and Giza1 cultivars, different responses were observed from the two cultivars. The higher growth of Titicaca cultivar in non-saline treatment is contrary to the results of previous researchers [16,21], who showed the optimal growth for different cultivars of quinoa to occur under 100 to 200 mM salinity stress. However, the higher growth of Giza1 cultivar in 21-dS m^−1^ salinity stress is in accordance with the results of previous research [20,22,23]. A relatively good tolerance was observed in both cultivars. One of the basic tolerance mechanisms for all plants, especially halophytes, is to maintain osmotic regulation through the accumulation of organic solutions, such as betaine, glycine, proline, and sorbitol. However, the production of these organic osmolytes to maintain osmotic and ionic regulation for the plant is associated with more energy consumption and thus reduces growth and yield [12,15,24].

The results of this study also showed that 0.75-mM SA concentration had the greatest effect on the modulation of the negative effect of salinity, which led to the highest dry weight of shoots and roots, whereas SA application at 1.5-mM concentration in some cases had a negative effect on plant growth. This increase in yield by SA may be due to its physiological roles, including ion uptake, and photosynthetic processes [17,25], which directly or indirectly regulate the function of plants. The optimum concentration of SA for most plants is between 0.5 to 1 mM and can cause tolerance to abiotic stress. Due to the role of SA in the redox regulation, its high concentration may reduce stress tolerance through disorder of the redox state [17].

The results showed with increasing salinity stress in both cultivars, the root dry weight was also decreased. Moreover, greater decrease in root dry weight of Titicaca than Giza1 can be attributed to the higher sensitivity of Titicaca to salt stress. One of the primary impacts of salinity on plants is to reduce the growth rate. By creating salinity and osmotic stress in the root growth environment, the roots are the first organ that faces salinity stress [26]. Due to osmotic regulation and avoidance mechanisms to salinity tolerance, a large amount of energy from the shoots is used in roots. It uses the air ions it receives for growth to deal with salinity stress. This behavior reduces the efficiency of the root in the absorption of nutrients and water compared to other organs of the plant, which consequently lead to root-growth loss [27]. The greater shoot and root dry weight in Giza1 at 21 dS m^−1^ could be attributed to higher salinity tolerance and the halophytic nature, some of which grow better in salinity conditions. These results are consistent with the results of a study that showed the growth of some Peruvian quinoa genotypes under salinity were decreased and were increased in some others [20].

External application of SA increased root growth of both quinoa cultivars in both saline and non-saline conditions. Previous reports have shown that treatment of plants with SA improves fresh and dry weight of shoots and roots under stress. For example, studies on various crops, including sunflower [28], bean [1], barley [25], and tomato [29,30], have shown that SA exogenous application increases root growth in both saline and non-saline conditions. Exogenous application of SA up to 0.75 mM was associated with greater root growth in Titicaca under non-saline conditions and in both cultivars under saline conditions. Accordingly, it can be concluded that the optimum SA concentration for quinoa root growth was 0.75 mM. Changing root morphology is one of mechanisms to improve salinity tolerance in quinoa. It has been reported that quinoa, instead of creating deep and dense root systems to modulate the negative impact of drought and find more water [16], prevents excessive Na^+^ and Cl^−^ absorption by reducing root growth and elongation [20]. Salicylic acid may help improve salinity tolerance of plants by altering root morphology, a hypothesis that requires careful testing.

Low concentrations of SA reduce the negative effect of salt stress. Application of SA increases plant growth and improves salinity tolerance through increased photosynthesis rate [30,31], enhanced indole acetic acid (IAA) levels, stimulated cell division and growth [29], stimulated the antioxidant system, and thus protection of cell membranes against oxidative stress and also by improving nutrient uptake [32].

Depending on its severity, salinity stress was associated with a decrease in K^+^ concentration and an increase in Na^+^ concentration in shoot so that the lowest K^+^ concentration and the highest Na^+^ concentration was obtained in the most severe salinity treatment. While salt stress enhanced concentration of both Na^+^ and K^+^ in roots, K/Na was decreased in shoot and roots of stressed plants. Salt stress disturbs the ion balance in the cytosol of the stressed plants. Under these conditions, the plant strategy is enhancing Na^+^ output and reducing K^+^ input to modulate the detrimental impact of salts. Conversely, increasing K^+^ concentration can reduce the effect of salinity on growth and yield [33]. Exposure of different quinoa cultivars to salinity stress resulted in accumulation of organic solutions (soluble sugar, proline, and protein), while in the leaves and roots, the amount of mineral ions (Na^+^ and K^+^) were increased, but the K/Na was decreased [20]. In quinoa, the removal of Na^+^ from cells and excessive storage in leaf vacuoles are an important protective mechanism in response to ion toxicity due to salinity stress at the cellular level. In addition, quinoa plants tolerate salinity by storing excess salt in epidermal cells on the leaves. It has been shown that the shape and size of epidermal cells change under salinity stress [34].

A significant inverse relationship was observed between tissue Na^+^ concentration with growth as well as with salinity tolerance in quinoa cultivars. The inverse relationship between Na^+^ accumulation in leaves and salinity tolerance often occurs when different genotypes in a species are compared but not in comparison between different species, such as wheat and barley [10]. On the other hand, the role of increasing K^+^ content in modulating the adverse effects of salinity stress is a complex process, which is mistaken for very simple and general. Interestingly, in a study on 11 different quinoa genotypes, a positive correlation was observed between the amount of accumulated Na^+^ and salinity tolerance of plants [24]. The negative correlation between leaf Na^+^ content and plant salinity tolerance suggests that the main effective mechanism for salinity tolerance is the removal of Na^+^ from the leaves [34].

In both studied cultivars, the accumulation of K^+^ was decreased under salinity stress. K^+^, as one of the most important and necessary ions for plant growth, is always needed as an enzyme cofactor and a vacuolar osmoticum. Thus, catalytic sites typically bind essential K and help plant growth under salinity stress by maintaining a high ratio of cytosolic K/Na [33]. As an indicator of salinity tolerance, the K/Na in vegetative tissues can be used as a suitable selection criterion for screening the quinoa genotypes [20]. Given the important physiological roles in plant cells, maintaining an adequate amount of K^+^ under salinity stress is very important. Therefore, the ability of plants to limit K^+^ loss and maintain more K^+^ than Na^+^ is directly related to plant salinity tolerance [13].

The application of SA could moderate a part of the negative effect of salinity on the ionic balance of K^+^ and Na^+^ concentrations in both shoot and root, i.e., increased shoot K^+^ and decreased root Na^+^ concentrations. Salicylic acid plays an important role in regulating the content of nutrients in plants. Various studies on different plants, including cucumber [32], sunflower [28], barley [25], and tomato [30], showed the use of SA increased the concentration of K^+^ in plants under saline and non-saline conditions. Treatment of mung bean plants with 0.5 mM SA resulted in a maximum reduction in Na^+^ and Cl^−^ concentration, while the levels of nitrogen, phosphorus, K^+^, and Ca^2+^were increased in both non-saline and saline conditions [31].

In line with Na^+^, the Cl^−^ concentration in shoot and root also increased in saline condition and with the increase in salinity levels. Na^+^ and Cl^−^ are two toxic ions that can cause significant disruptions in plant biological processes. In this study, the concentration of Na^+^ was increased much more than Cl^−^ in saline conditions. In addition, high concentrations of NaCl in the soil cause high accumulation of these ions in plants. In fact, Na^+^ and Cl^−^ homeostasis is essential for plants to maintain active growth in saline conditions. Moreover, increasing the amount of Na^+^ and Cl^−^ with increasing salinity levels in other plants, such as sunflower [28], beans [1], barley [25], and tomato [29,30], has also been reported. Under saline conditions, the increase in Na^+^ and Cl^−^ in plant tissues occurs due to the entry of high ions through non-selective cationic and anionic channels. In addition, the accumulation of Na^+^ and Cl^−^ in the roots disrupts the mechanism of nutrient uptake by the cell membrane, thereby increasing the transport of Na^+^ and Cl^−^ to the shoots [10]. It has also been shown that inactive Cl^−^ uptake is increased with depolarization of membrane potential and low amount of intracellular Cl^−^ under saline conditions [8]. In plants exposed to high salinity, the accumulation of toxic ions, such as Na^+^ and Cl^−^, in chloroplasts inhibits electron transfer and photophosphorylation of the thylakoid membrane [25].

There are different mechanisms for salinity tolerance among various quinoa genotypes. For example, Shabala et al. [24] showed that three of the 14 salt-tolerant quinoa genotypes stored small amounts of Na^+^ and therefore had a Na^+^-excretion mechanism. The remaining 11 genotypes accumulated relatively large amounts of Na^+^ in vacuoles, indicating that the common mechanism of salinity tolerance in quinoa is Na^+^ compartmentation. In our research, it seemed that the two quinoa cultivars had two different salinity-tolerance mechanisms.

The SA foliar application could reduce a part of the enhanced Cl^−^ concentration due to salinity in both quinoa cultivars, and this effect was intensified by increasing the SA concentrations. That is, the lowest Cl^−^ concentration was obtained at the highest SA concentration (i.e., 1.5 mM). Typically, exogenous application of SA enhanced the concentrations of K^+^, Ca^2+^, Mg^2+^, iron, manganese, copper, phosphorus, nitrogen, and sulfur in the tissues of many plant species but minimizes the absorption of Na^+^ and Cl^−^ [32]. Decreased absorption of Na^+^ and Cl^−^ and consequent reduction of toxicity of these two ions in plants treated with SA can be a symbol of relative salinity tolerance [31]. It seems that the reduction of Na^+^ and Cl^−^ through the application of SA under salinity stress can be due to the reduction of damage to cell membranes by stimulating the activity of antioxidant enzymes or due to the dilution effect resulting increased dry matter production [1,28].

Increasing salinity to moderate levels (7 dS m^−1^) increased the concentrations of Ca^2+^ and Mg^2+^ in shoot and root, and more severe stress levels decreased the concentrations of these two ions. Ca^2+^ and Mg^2+^ are secondary nutrients for plant growth. Ca^2+^ is responsible for maintaining the cell walls of plants and is used to activate specific enzymes and to send signals to coordinate cellular activity [35]. Magnesium plays an important role in photosynthesis as a part of the chlorophyll structure and a cofactor for photosynthetic enzymes. In addition, Mg^2+^ contributes to the stability of various macromolecules, including proteins, cell wall and membrane, maintenance of enzymatic activity, and homeostasis of reactive oxygen species under saline conditions [17,26].

In this study, the lowest amount of Ca^2+^ and Mg^2+^ were obtained in 21dS m^−1^ salinity stress. Decreases in Ca^2+^ and Mg^2+^ content at high salinity levels have been reported in various plants, as nutrient uptake by roots is difficult in saline soils. Panda et al. [9] observed a similar result about the reduction of element uptake in halophyte species *Suaeda maritima* and *Atriplex atacamensis* under salinity stress. Since the uptake and transfer of elements depends on the transpiration rate of plants, it seems that a part of this reduction is due to reduced transpiration of plants and low root pressure under salinity stress [13]. The decrease in available Ca^2+^ under salinity is also related to the displacement of Na^+^ by Ca^2+^ at extracellular junctions [26]. Surprisingly, Mg^2+^ concentration in shoots and roots and its SF correlated more than expected with salinity tolerance.

Salicylic acid had a positive effect on the absorption of Ca^2+^ and Mg^2+^ ions; this probably reduced some of the negative effects of salinity stress. This effect may be related to the role of SA in reducing the absorption of Na^+^ and increasing root growth; however, other roles of SA in physiological processes may also play a part. There is an interaction between Na^+^ and Ca^2+^ in plants, which has a great impact on cell membrane properties and ion transport. Sodium also reduces Mg^2+^ content in plants by preventing its transfer to young leaves through the phloem (rinsing vessel) as well as the formation of ion pairs [17]. The ability to limit the entry of toxic ions, such as Na^+^ and Cl^−^, into root cell membranes as a result of the exogenous SA application is likely increased through its signaling role [30].

Salinity 7 dS m^−1^ increased the K^+^ to Na^+^ selectivity; however, salinities 14 and 21 dS m^−1^ sharply reduced it. The selectivity index was also significantly greater in the SA-treated plants in both cultivars but was significantly more in Titicaca than Giza1 cultivar. As a general reaction to salinity tolerance, plants retain more Na^+^ in their roots and limit its transport to shoots. This behavior is due to relatively higher tolerance to ion toxicity of roots than leaves. Compared to the root, the very low amount of Na^+^ in the leaves of quinoa cultivars indicates the mechanism of Na^+^ accumulation in the roots or the removal from the leaves [24]. Under salinity conditions, exchange takes place between Na^+^ and K^+^ close to the root, and this K^+^ is transferred to the leaves as it is released into the xylem [33]. Protecting young leaves from excessive amounts of Na^+^ has been well known as one of the most important salinity tolerance mechanisms [10,34]. Quinoa also seems to follow this mechanism.

The change in SF showed a clear trend for the ions, including Na^+^, Cl^−^, and Ca^2+^. Salinity, in general, increased the SF of all ions, which was higher in Na^+^, Cl^−^, and K^+^. This means that under saline conditions, the plant firstly absorbs more soluble ions from the soil and secondly retains more ions in the roots and does not transfer them to the shoot. This suggests that one of the basic strategies of quinoa to deal with salinity stress is the storage of toxic ions, such as Na^+^ and Cl^−^, in the roots [35]. The results of Pirasteh-Anosheh and Emam [25] showed that salinity increased and decreased the KSF and NaSF, respectively; this indicated that in stressed barley plants, higher amounts of Na^+^ and lower amounts of K^+^ were transferred to the shoots, which in turn reduced growth. They also reported that NaSF was increased in plants treated with SA. Roots are more tolerant to salt stress than leaves, and the transfer of toxic Na^+^ and Cl^−^ ions to the shoot may be the main reason for reduced shoot dry weight in saline conditions [29], which also was observed in the current research. It has been reported that in saline conditions, greater amounts of K^+^, Ca^2+^, and Mg^2+^ are absorbed by the roots, and transmission to the shoot is limited [27]. Salinity stress reduces K^+^ uptake and transfer into shoots by selecting in favor of Ca^2+^ uptake [28]. It has been argued that selectivity in favor of K^+^ may be reduced in saline conditions [27]. The results of correlation and regression analyses better reflected the greater importance of the K/Na than the concentration of the individual ions. Furthermore, ion distribution was more important than ion accumulation in order to keep optimal growth under saline conditions.

## 5. Conclusions

Salinity tolerance of the two quinoa cultivars was different: Giza1 had more growth and tolerance, while both cultivars uniformly responded to exogenous SA. The concentration of all ions in roots was higher than shoots, which indicates a higher accumulation of salts in roots for better salinity tolerance. The changes in selectivity index and SF revealed SA could change the ion distribution in favor of the plant; i.e., less Na^+^ and Cl^−^ and more Ca^2+^ and Mg^2+^ were transferred to shoots. Interestingly, this mechanism was more tangible in the Giza1, as the more tolerant cultivar. Therefore, it can be clearly claimed that one of the most important mechanisms of salinity tolerance in quinoa was the accumulation of salts in the roots to prevent damage to the cytosol in the shoot. More research is needed on the salts compartmentation, especially transferring Na^+^ and Cl^−^ to shoots in different shoot organelles. The importance of Mg^2+^ in salinity tolerance of quinoa and maybe other plants has been underestimated.

## Figures and Tables

**Table 1:** The concentrations of ions (%) in quinoa shoots treated with varied salicylic acid (SA) concentrations grown under different salinity levels.

Cultivar	Salt Stress (dS m^−1^)	SA Level (mM)	Na^+^		K^+^		Cl^─^		Ca^2+^		Mg^2+^		K/Na	
Giza1	0	0	0.72	±0.064	0.86	±0.070	0.63	±0.023	1.33	±0.062	0.74	±0.117	1.20	±0.094
		0.75	0.39	±0.099	1.00	±0.062	0.42	±0.008	1.61	±0.096	0.98	±0.234	2.93	±0.766
		1.5	0.35	±0.091	1.01	±0.064	0.40	±0.008	1.71	±0.099	0.92	±0.224	3.37	±0.911
	7	0	1.38	±0.142	0.64	±0.073	0.90	±0.057	1.49	±0.095	1.13	±0.233	0.47	±0.054
		0.75	0.92	±0.092	0.73	±0.048	0.69	±0.038	1.70	±0.117	1.23	±0.309	0.81	±0.105
		1.5	0.84	±0.094	0.73	±0.050	0.71	±0.037	1.73	±0.104	1.19	±0.293	0.90	±0.113
	14	0	2.51	±0.108	0.42	±0.066	1.08	±0.077	1.16	±0.129	1.09	±0.376	0.17	±0.022
		0.75	1.92	±0.208	0.57	±0.079	0.90	±0.076	1.47	±0.157	1.01	±0.235	0.31	±0.057
		1.5	1.89	±0.180	0.56	±0.082	0.89	±0.060	1.43	±0.162	0.97	±0.222	0.31	±0.052
	21	0	2.66	±0.318	0.39	±0.059	1.29	±0.103	1.01	±0.082	0.68	±0.028	0.15	±0.021
		0.75	2.01	±0.156	0.50	±0.067	1.01	±0.082	1.35	±0.110	0.95	±0.185	0.26	±0.044
		1.5	2.09	±0.163	0.50	±0.067	0.99	±0.076	1.21	±0.061	0.81	±0.071	0.24	±0.044
Titicaca	0	0	0.77	±0.038	0.83	±0.098	0.64	±0.014	1.35	±0.078	0.90	±0.161	1.09	±0.153
		0.75	0.43	±0.069	0.96	±0.102	0.43	±0.011	1.62	±0.088	1.21	±0.250	2.31	±0.266
		1.5	0.38	±0.065	0.96	±0.106	0.41	±0.011	1.76	±0.109	1.20	±0.291	2.62	±0.335
	7	0	1.48	±0.090	0.61	±0.101	0.92	±0.038	1.53	±0.102	1.39	±0.271	0.42	±0.083
		0.75	1.01	±0.077	0.68	±0.082	0.71	±0.022	1.67	±0.107	1.50	±0.305	0.70	±0.137
		1.5	0.92	±0.069	0.69	±0.084	0.72	±0.022	1.68	±0.104	1.44	±0.300	0.77	±0.148
	14	0	2.66	±0.174	0.40	±0.090	1.10	±0.054	1.32	±0.280	1.38	±0.348	0.15	±0.035
		0.75	2.08	±0.194	0.53	±0.114	0.92	±0.056	1.49	±0.150	1.22	±0.233	0.26	±0.065
		1.5	2.07	±0.179	0.52	±0.114	0.92	±0.037	1.65	±0.244	1.31	±0.324	0.26	±0.071
	21	0	2.84	±0.193	0.36	±0.083	1.33	±0.066	1.12	±0.184	0.85	±0.187	0.13	±0.033
		0.75	2.21	±0.180	0.46	±0.097	1.05	±0.050	1.48	±0.173	1.22	±0.257	0.22	±0.061
		1.5	2.30	±0.181	0.46	±0.097	1.03	±0.047	1.25	±0.098	0.98	±0.171	0.21	±0.058

±Standard error (SE).

**Table 2:** The concentrations of ions (%) in quinoa roots treated with varied salicylic acid (SA) concentrations grown under different salinity levels.

Cultivar	Salt Stress (dS m^−1^)	SA Level (mM)	Na^+^		K^+^		Cl^−^		Ca^2+^		Mg^2+^		K/Na	
Giza1	0	0	0.17	±0.009	1.01	±0.097	0.69	±0.057	1.29	±0.08	0.62	±0.03	5.87	±0.38
		0.75	0.18	±0.010	1.06	±0.098	0.65	±0.041	1.26	±0.07	0.68	±0.03	5.71	±0.35
		1.5	0.20	±0.011	1.10	±0.099	0.62	±0.032	1.19	±0.05	1.30	±0.03	5.48	±0.31
	7	0	1.18	±0.145	2.21	±0.149	0.84	±0.060	1.61	±0.11	1.44	±0.06	1.90	±0.15
		0.75	1.58	±0.150	2.62	±0.176	0.96	±0.050	1.62	±0.08	2.07	±0.06	1.67	±0.09
		1.5	2.14	±0.112	2.79	±0.187	1.35	±0.036	1.45	±0.09	1.75	±0.06	1.30	±0.03
	14	0	1.71	±0.088	2.56	±0.172	1.31	±0.141	1.37	±0.06	1.33	±0.06	1.50	±0.05
		0.75	2.97	±0.206	3.01	±0.202	1.43	±0.075	1.36	±0.06	1.84	±0.06	1.01	±0.02
		1.5	3.37	±0.191	2.90	±0.188	1.90	±0.090	1.21	±0.02	1.16	±0.05	0.86	±0.01
	21	0	2.35	±0.218	3.08	±0.207	1.71	±0.270	0.88	±0.01	0.58	±0.04	1.32	±0.06
		0.75	3.58	±0.107	3.31	±0.223	2.03	±0.106	0.97	±0.03	1.31	±0.05	0.92	±0.04
		1.5	4.16	±0.125	3.71	±0.149	2.35	±0.158	0.82	±0.01	0.94	±0.05	0.89	±0.04
Titicaca	0	0	0.34	±0.023	0.48	±0.033	0.74	±0.023	1.18	±0.072	0.424	±0.041	1.44	±0.003
		0.75	0.33	±0.022	0.45	±0.030	0.77	±0.040	1.25	±0.067	0.532	±0.043	1.35	±0.005
		1.5	0.35	±0.027	0.42	±0.028	0.83	±0.036	1.05	±0.067	0.639	±0.045	1.18	±0.014
	7	0	1.31	±0.050	1.28	±0.086	0.91	±0.038	1.32	±0.055	0.456	±0.037	0.99	±0.097
		0.75	1.45	±0.030	0.78	±0.053	1.12	±0.059	1.41	±0.073	0.434	±0.032	0.54	±0.026
		1.5	2.51	±0.101	0.54	±0.036	1.40	±0.111	1.19	±0.015	0.431	±0.036	0.22	±0.017
	14	0	1.64	±0.042	2.15	±0.145	1.12	±0.067	1.12	±0.070	0.150	±0.037	1.32	±0.105
		0.75	2.11	±0.039	1.82	±0.122	1.71	±0.010	1.17	±0.077	0.825	±0.043	0.86	±0.043
		1.5	3.57	±0.025	1.29	±0.087	2.07	±0.031	0.98	±0.021	0.635	±0.037	0.36	±0.024
	21	0	2.09	±0.104	1.77	±0.119	1.53	±0.053	0.81	±0.020	0.107	±0.037	0.85	±0.071
		0.75	2.54	±0.051	1.89	±0.127	2.20	±0.054	0.87	±0.012	0.889	±0.043	0.75	±0.057
		1.5	3.86	±0.078	1.28	±0.086	2.40	±0.062	0.73	±0.006	0.899	±0.045	0.33	±0.028

±Standard error (SE).

**Table 3:** The storage factor of ions in quinoa plants treated with varied salicylic acid (SA) concentrations grown under different salinity levels.

Cultivar	Salt Stress (dS m^−1^)	SA Level (mM)	Na^+^		K^+^		Cl^−^		Ca^2+^		Mg^2+^	
Giza1	0	0	0.192	±0.006	0.540	±0.033	0.525	±0.012	0.491	±0.023	0.455	±0.045
		0.75	0.319	±0.054	0.513	±0.032	0.609	±0.012	0.438	±0.015	0.410	±0.062
		1.5	0.365	±0.059	0.521	±0.032	0.607	±0.010	0.410	±0.015	0.586	±0.062
	7	0	0.461	±0.033	0.774	±0.011	0.481	±0.021	0.519	±0.020	0.561	±0.047
		0.75	0.633	±0.028	0.783	±0.011	0.580	±0.002	0.488	±0.016	0.626	±0.054
		1.5	0.719	±0.013	0.792	±0.011	0.657	±0.006	0.456	±0.018	0.596	±0.056
	14	0	0.405	±0.021	0.859	±0.013	0.548	±0.010	0.542	±0.036	0.549	±0.070
		0.75	0.608	±0.029	0.841	±0.013	0.614	±0.010	0.480	±0.024	0.644	±0.048
		1.5	0.641	±0.024	0.838	±0.016	0.681	±0.008	0.459	±0.024	0.543	±0.052
	21	0	0.469	±0.025	0.888	±0.011	0.571	±0.025	0.466	±0.022	0.461	±0.013
		0.75	0.640	±0.021	0.869	±0.012	0.667	±0.007	0.418	±0.024	0.579	±0.042
		1.5	0.666	±0.022	0.882	±0.010	0.703	±0.021	0.403	±0.010	0.537	±0.019
Titicaca	0	0	0.305	±0.024	0.372	±0.037	0.538	±0.009	0.466	±0.027	0.331	±0.057
		0.75	0.443	±0.054	0.321	±0.030	0.641	±0.016	0.436	±0.027	0.321	±0.061
		1.5	0.488	±0.060	0.306	±0.029	0.669	±0.014	0.374	±0.030	0.368	±0.074
	7	0	0.469	±0.007	0.679	±0.042	0.496	±0.021	0.463	±0.026	0.261	±0.052
		0.75	0.593	±0.023	0.537	±0.035	0.610	±0.018	0.457	±0.029	0.239	±0.049
		1.5	0.733	±0.013	0.443	±0.036	0.656	±0.025	0.416	±0.016	0.246	±0.053
	14	0	0.382	±0.016	0.846	±0.031	0.504	±0.013	0.469	±0.056	0.116	±0.048
		0.75	0.505	±0.027	0.777	±0.039	0.651	±0.014	0.442	±0.041	0.416	±0.057
		1.5	0.635	±0.020	0.716	±0.047	0.694	±0.007	0.381	±0.041	0.348	±0.070
	21	0	0.425	±0.021	0.832	±0.033	0.536	±0.004	0.428	±0.033	0.128	±0.051
		0.75	0.537	±0.017	0.806	±0.033	0.677	±0.016	0.375	±0.026	0.435	±0.062
		1.5	0.628	±0.014	0.739	±0.043	0.701	±0.015	0.370	±0.016	0.486	±0.052

±Standard error (SE).

**Table 4:** Correlation of dry weight with ions accumulation and distribution.

	Sodium			Potassium			K/Na Ratio		K/Na Selectivity
	Shoot	Root	SF	Shoot	Root	SF	Shoot	Root	
Shoot dry weight	−0.261 ns	−0.03 ns	0.082 ns	0.278 ns	0.396 *	−0.531 **	0.433 *	0.413 *	−0.159 ns
Root dry weight	−0.407 *	−0.217 ns	−0.138 ns	0.372 ns	−0.097 ns	0.038 ns	0.426 *	0.242 ns	0.047 ns
Total dry weight	−0.405 *	−0.147 ns	0.381 *	0.335 ns	0.410 *	−0.058 ns	0.624 **	0.440 *	−0.095 ns
	**Chlorine**			**Calcium**			**Magnesium**		
	**Shoot**	**Root**	**SF**	**Shoot**	**Root**	**SF**	**Shoot**	**Root**	**SF**
Shoot dry weight	−0.265 ns	−0.067 ns	0.403 *	0.172 ns	0.081 ns	−0.062 ns	−0.485 **	0.473 *	0.526 **
Root dry weight	−0.352 ns	−0.120 ns	0.220 ns	0.131 ns	−0.094 ns	−0.208 ns	−0.149 ns	−0.023 ns	0.075 ns
Total dry weight	−0.225 ns	−0.113 ns	0.452 *	0.221 ns	0.052 ns	−0.144 ns	−0.402 *	0.409 *	0.444 *

SF, storage factor; ns, not significant; * and ** significant at 5% and 1% probability levels.

**Table 5:** Stepwise regression analysis between dry weight and ions accumulation and distribution.

	Variable Entered	Partial R^2^	Model R^2^	F Value	Pr > F
Total dry weight	MgSF	0.703	0.703	52.28	0.001
	Shoot Mg^2+^	0.190	0.893	32.17	0.003
	Shoot K/Na	0.050	0.943	16.50	0.022
	Root K/Na	0.030	0.973	11.90	0.012
	ClSF	0.019	0.992	9.25	0.043
Root dry weight	Root K/Na	0.882	0.882	66.67	0.001
	Shoot Na^+^	0.092	0.974	20.91	0.011
	NaSF	0.021	0.995	10.75	0.022

## Data Availability

The datasets generated and/or analysed during the current study are available from the corresponding author upon reasonable request.
